# Tai Chi Chuan Exercise Improves Lung Function and Asthma Control through Immune Regulation in Childhood Asthma

**DOI:** 10.1155/2019/9146827

**Published:** 2019-10-23

**Authors:** Pei-Chun Liao, Han-Hong Lin, Bor-Luen Chiang, Jyh-Hong Lee, Hsin-Hui Yu, Yu-Tsan Lin, Yao-Hsu Yang, Pei-Yi Li, Li-Chieh Wang, Wei-Zen Sun

**Affiliations:** ^1^Department of Pediatrics, National Taiwan University Hospital, Taipei, Taiwan; ^2^Graduate Institute of Networking and Multimedia, College of Electrical Engineering and Computer Science, National Taiwan University, Taipei, Taiwan; ^3^Department of Medical Research, National Taiwan University Hospital, Taipei, Taiwan; ^4^Taiwan Tai Chi Academy, Taipei, Taiwan; ^5^Department of Anesthesiology, National Taiwan University Hospital, Taipei, Taiwan

## Abstract

**Background:**

Tai Chi Chuan (TCC) is an exercise of low to moderate intensity with key features of mindfulness, structural alignment, and flexibility to relax the body and mind in adults. Our previous study showed that TCC could improve the quality of life (QoL), pulmonary function, and fractional exhaled nitric oxide in asthmatic children. We further investigated whether the benefits induced by TCC were associated with immune regulation.

**Method:**

Six- to twelve-year-old children diagnosed with mild to severe persistent asthma for at least one year according to the Global Initiative for Asthma guidelines were enrolled from a tertiary pediatric allergy center in Taiwan. Asthmatic children were divided into two groups based on their choice: (1) the TCC group had a 60-minute TCC exercise session once weekly led by an instructor and (2) the control group kept their original activity levels. All other exercises were encouraged as usual. Pulmonary function tests, laboratory tests, standardized pediatric asthma QoL questionnaire (PAQLQ(S)), and childhood asthma control test (C-ACT) were performed before and after the TCC program (12 weeks). Data on medications and exacerbations were collected from medical records.

**Results:**

There were no differences between the TCC (*n* = 25) and control (*n* = 15) groups at baseline, except that the C-ACT showed significantly lower results in the TCC group (*p*=0.045). After 12 weeks, the number of leukocytes (*p*=0.041) and eosinophils (*p*=0.022) decreased, while regulatory T cells increased significantly (*p*=0.008) only in the TCC group. Lung functions (FEV_1_ and PEFR) were significantly improved in both the TCC (*p* < 0.001) and control (*p*=0.045 and 0.019, respectively) groups, while the PAQLQ(S) and C-ACT (*p* < 0.001) showed improvement only in the TCC group. Moreover, compared to the control group, the exacerbations within 12 weeks after the study were significantly decreased in the TCC group (*p*=0.031). After multiple regression by a conditional forward method, the factors that were significantly associated with exacerbation within 12 weeks after study is the practice of TCC and exacerbation within 24 weeks before study (*p*=0.013 and 0.015, respectively) after adjusting for age, sex, asthma severity, PEF, FEV_1_, C-ACT, PAQLQ(S), and medication score at baseline.

**Conclusion:**

TCC exercise may improve pulmonary functions, asthma control, and QoL and prevent exacerbations in asthmatic children through immune regulation. Further research on detailed mechanisms is mandated.

## 1. Introduction

Asthma, one of the major chronic airway diseases in children, remains a leading health concern in all parts of the world. The increase in childhood asthma prevalence has been noted in Taiwan in recent years [1, 2]. This rising trend imposes significant societal and economic burdens, resulting in missed school/work days, activity limitations, and increased healthcare utilization.

An important aspect in asthmatic care is the level of control among asthmatic patients since the most expensive component of asthmatic care is related to acute care, including emergency visits and hospitalization [3]. Sun et al. reported that pediatric patients with asthma used substantially more healthcare services and required higher medical costs than patients without asthma [4]. It is evident that asthmatic care still imposes a large economic burden on patients' families in Taiwan.

The primary management for asthma is medications supplemented with exercise [5]. In a review of the impact of exercise on asthma, common types of exercise, such as walking, jogging, swimming, and cycling, are generally very safe for children and adults with asthma. Exercise appears to favor improvements in aerobic fitness, asthma symptoms, and quality of life (QoL) but results so far are less consistent in demonstrating improvements in lung function and airway hyperresponsiveness [6]. However, exercise‐induced bronchoconstriction presented in 52.5% of children with asthma [7] and exercise should be done with caution in unstable asthmatic patients. Tai Chi Chuan (TCC), unlike aerobic exercise, is a form of mind and body exercise that employs detailed regimens of flowing circular movements, balance and weight shifting, breathing techniques, and cognitive visualization with focused internal awareness [8]. Studies have investigated the effects of TCC as an intervention for a wide variety of health concerns and demonstrated benefits for cardiopulmonary functions, mental stability, body flexibility, and balance control [9–11]. TCC was also proven to have anti-inflammatory effects, including reducing monocyte counts, enhancing the CD4^+^/CD8^+^ T cell ratio, and increasing the amount of regulatory T (Treg) cells [12, 13]. Thus, TCC, a mild-to-moderate-intensity exercise free of space and weather restrictions, provides an ideal option for asthmatic children.

Asthma is characterized by T helper cell 2 (Th2) type inflammation, leading to airway hyperresponsiveness and tissue remodeling [14]. Treg cells have a key role in promoting and maintaining tolerance to allergens by regulating both innate and adaptive allergen-triggered immune responses. In addition to their immunosuppressive functions and capacities to restrict the intensity of immune responses, Treg cells can also control nonimmunological processes, such as tissue repair, resulting from extensive inflammation [15]. Treg cells are key players in maintaining pulmonary homeostasis and airway tolerance, and through manipulating these, Treg cell-involved pathways may provide more effective therapy to treat asthmatic individuals [14]. In our previous small-scale studies, the potential of a 12-week TCC training program on pulmonary function improvement in asthmatic children was demonstrated [16, 17]. We also found that compared to the control group, asthmatic children who performed 60 minutes of TCC exercise once weekly for 12 weeks had significantly decreased fractional exhaled nitric oxide (FeNO), increased forced expiratory volume in one second/forced vital capacity (FEV_1_/FVC), and improved QoL [17]. Since an increasing amount of Treg cells was noted in TCC [12], we speculate that these increasing Tregs may suppress airway inflammation and improve lung functions in asthma patients. In this study, we aimed to investigate the mechanism of action of TCC on asthmatic children. Apart from the clinical control, medications, exacerbations, and lung function tests, we also explored the possible biochemical significance and evidence of the role of Treg cells in modulating airway inflammation following TCC exercise.

## 2. Materials and Methods

### 2.1. Study Subjects and Study Design

Six- to twelve-year-old children diagnosed with mild to severe persistent asthma for at least one year according to the Global Initiative for Asthma (GINA) guidelines for 2012 [18] were enrolled from an outpatient pediatric allergy clinic at National Taiwan University Hospital (NTUH), Taiwan. Subjects with congenital heart disease, chronic cardiopulmonary disease, autoimmune disease, and neuromuscular disease were excluded. The study was approved by the Ethics Committee of NTUH and informed consent was obtained from both the participant and their legal guardian.

At enrollment, participants were divided into two groups based on their choice: (1) the TCC group had a 60-minute TCC exercise session once weekly led by an instructor and (2) the control group kept their original activity levels. The intervention period lasted for 12 weeks, from July 12 to October 4, 2014. Exercise adherence was monitored by the instructor, and attendance was recorded by the project assistant. In addition to the once weekly training session, participants were asked to practice TCC exercise daily by following a video. Participants in the control group were instructed not to begin any new exercise and maintained only the original activity forms and levels. All participants continued to receive their regular medical care and medications during the study.

The TCC training course was specifically designed as therapy for asthmatic children. All program components were derived from Chen-style TCC standardized movements under the guidance of two TCC instructors (Li PY and Sun CH). The core training incorporated a sequence of warm-up circular movements, stretching exercises, TCC walking drills, and TCC “opening and closing” movements in stationary positions as well as breathing techniques.

### 2.2. Demographics and Laboratory Tests

Demographics including allergy history and body weight were obtained at enrollment. Laboratory tests including lung function tests, FeNO, blood cell counts, IgE levels, and asthmatic symptom surveys were conducted at baseline and 12 weeks after TCC training. Data of medications and exacerbations were collected from the participants' medical records. The severity of asthma was classified according to the GINA guidelines of 2012 [18]. Medication scores were calculated based on the weighting coefficient of the medication: 1 puff of 200 *μ*g fenoterol was scored 1 point, 1 puff of 50 *μ*g fluticasone propionate was scored 1 point, and 1 tablet of 5 mg prednisolone was scored 18 points. An asthma exacerbation was defined by increased coughing, chest tightness, or wheezing in association with an unscheduled visit, and/or oral prednisolone, and/or increased consumption of fenoterol by at least 50% for over more than two consecutive days.

#### 2.2.1. Lung Function Test

Pulmonary functions were measured using a Super Spiro spirometer (Micro Medical Ltd, UK) at rest. None of the participants had taken any short-acting bronchodilator four hours before spirometry. Participants in both groups were required to record a daily peak expiratory flow (PEF).

#### 2.2.2. FeNO

All subjects underwent measurement of FeNO using a NObreath® machine (Bedfont Scientific Ltd, UK) with standardized techniques published by the American Thoracic Society and European Respiratory Society [19].

#### 2.2.3. Standardized Pediatric Asthma Quality of Life Questionnaire (PAQLQ(S))

The validated Chinese version of the PAQLQ(S) [20] was used to analyze the QoL of pediatric asthmatics in this study. A change in score greater than 0.5 points on the 7-point scale was considered clinically significant [20, 21].

#### 2.2.4. Childhood Asthma Control Test (C-ACT)

The level of asthma control was measured using the Chinese version of the C-ACT [22, 23]. Scores greater than or equal to 20 indicated good asthma control, whereas scores less than or equal to 19 indicated inadequate asthma control in children.

#### 2.2.5. Allergen Sensitization

The total serum IgE level was measured using an ImmunoCAP assay (Phadia, Uppsala, Sweden). Specific IgE antibodies against common allergens among individual pediatric asthmatics were measured using a MAST Optigen allergy system (Hitachi Chemical Diagnostics, Mountain View, CA). Positive allergen sensitization was defined as a cutoff specific IgE value of 143LU or greater.

#### 2.2.6. Flow Cytometry Analysis of Treg Cells

Peripheral blood mononuclear cells (PBMCs) were isolated from heparinized blood by Ficoll-Hypaque (GE Healthcare, Buckinghamshire, UK) density gradient centrifugation (400*g* for 30 minutes at room temperature) and washed with cold PBS buffer (containing 2% FCS and 0.1% sodium azide). The cells were then stained with either anti-CD4-FITC and anti-CD25-PE monoclonal antibodies (Becton Dickinson, San Jose, CA) or isotype-matched controls for 30 minutes on ice. After being properly washed with the cold buffer, the stained cells were further incubated in a freshly prepared fixation/permeabilization working solution for 30 minutes on ice. After washing with permeabilization buffer twice, the stained cells were conjugated with either Foxp3-APC monoclonal antibodies (Becton Dickinson, San Jose, CA) or isotype-matched controls for another 30 minutes on ice. After proper washing, the cells were resuspended in a cold buffer and analyzed using a FACSort cell analyzer (Becton Dickinson). More than 2 × 10^4^ cells were analyzed for each sample, and the results were processed by Cellquest software (Becton Dickinson).

### 2.3. Statistical Analysis

Data between the TCC and control groups were compared using the Mann–Whitney *U* test for continuous variables and Pearson's chi square test for nominal variables. The Wilcoxon signed rank test was used to compare continuous variables before and after TCC. Multiple regression analysis by a conditional forward method was used to determine the significant factors for exacerbation within 12 weeks after the study. All statistical analyses were performed with IBM SPSS statistical software (version 20.0.0, IBM Corp., Armonk, NY). A *p* value of <0.05 was considered statistically significant.

## 3. Results

### 3.1. Study Population

**Figure 1:**
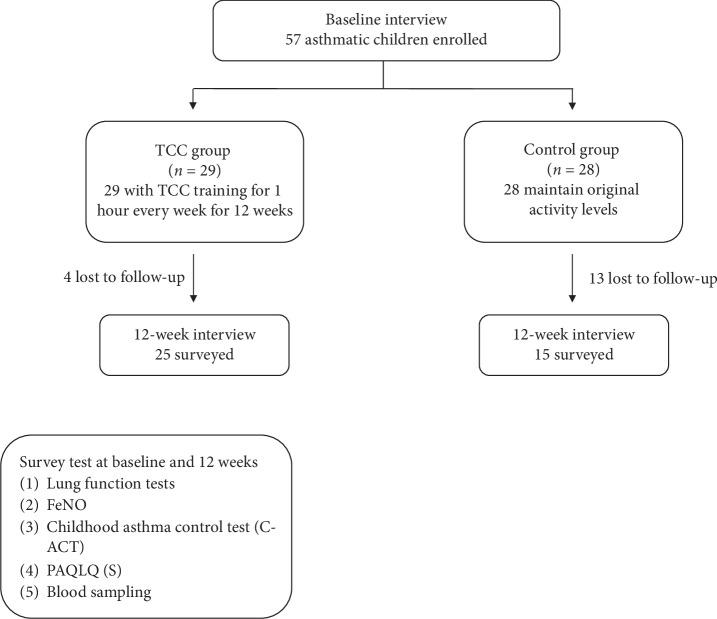
A flowchart of data collection.

Initially, 57 asthmatic children were enrolled in the study for the TCC group (*n* = 29) and control group (*n* = 28). However, 4 participants in the TCC group and 13 in the control group were lost to follow-up during the 12-week period (Figure 1). Not only the demographic data (e.g., sex, age, body weight, atopy history, asthma severity, medication scores, and exacerbations in the past 6 months) (Table 1) but also the laboratory and lung function tests at baseline showed no significant difference between the two groups except for the C-ACT results (Table 2), suggesting that both groups had similar baseline statuses, except that asthma control in the TCC group was poorer. Asthma severity was heterogeneous, although not significantly different, as the control group had a higher proportion of patients with severe and mild persistent asthma than the TCC group (26.7% vs. 16% and 60% vs. 52%, respectively) but a lower proportion with moderate persistent asthma (13.3% vs. 32%). The total IgE levels were not different between the two groups (Table 2). For the allergen sensitization test, the majority of patients in both the TCC and control groups were allergic to mites (70.8% and 66.7%, respectively). Other allergens detected were cockroaches (4.2% and 6.7%, respectively), cat dander (4.2% and 0%, respectively), and shellfish (8.3% and 6.7%, respectively).

### 3.2. Outcome Measurements

#### 3.2.1. Change in Peripheral Blood Cells

**Figure 2:**
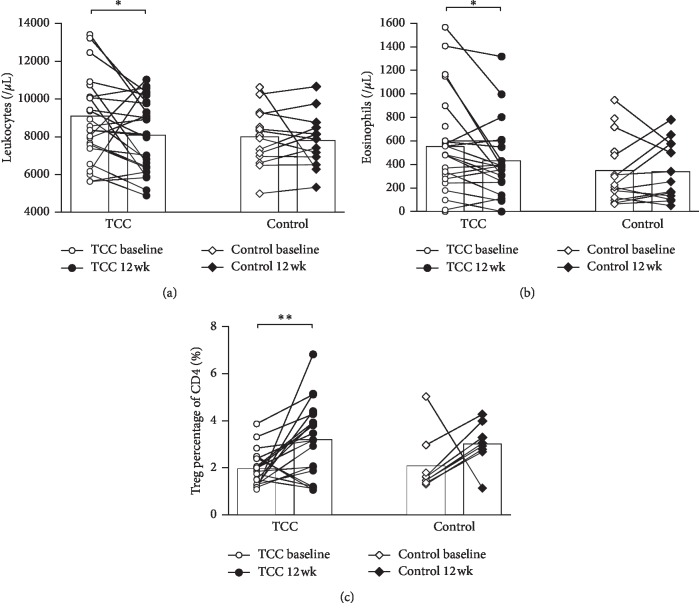
The amount of (a) leukocytes, (b) eosinophils, and (c) Treg cells in peripheral blood at baseline and 12 weeks after TCC. The bar denotes the mean level. ^*∗*^*p* < 0.05; ^*∗∗*^*p* < 0.01.

Significant decrease in total leukocytes and eosinophils (*p*=0.041 and 0.022, respectively) was observed only in the TCC group for paired data at baseline and after 12 weeks by the Wilcoxon signed rank test (Figures 2(a) and 2(b)). In peripheral blood, the percentage of CD4^+^ CD25^+^ Foxp3^+^ Treg cells in CD4^+^ T cells increased significantly (*p*=0.008) after 12 weeks of TCC exercise (Figure 2(c)).

#### 3.2.2. Improvement in Lung Functions

**Figure 3:**
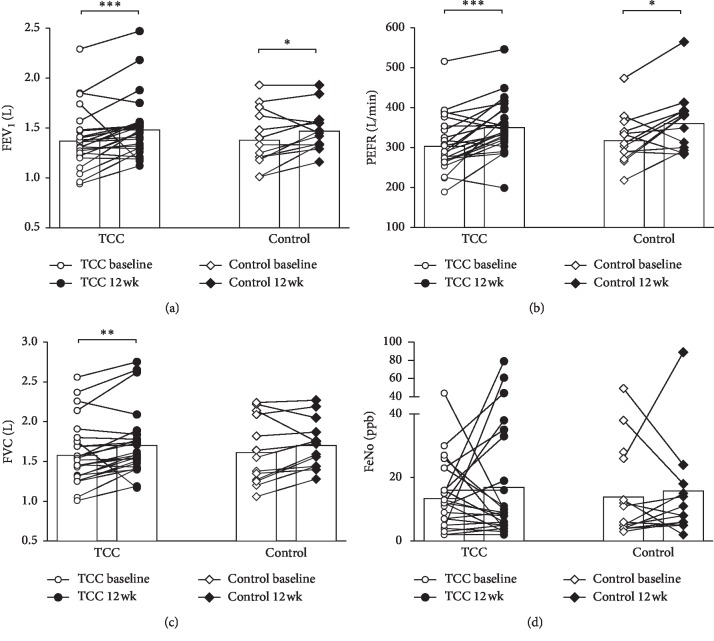
The results of lung function tests at baseline and 12 weeks after TCC. The bar denotes the mean level. ^*∗*^*p* < 0.05; ^*∗∗*^*p* < 0.01; ^*∗∗∗*^*p* < 0.001.

The TCC group had significantly improved FVC after 12 weeks compared to baseline (*p*=0.002) (Figure 3(c)). The levels of FEV_1_ and PEFR increased significantly in both TCC (*p* < 0.001) and control groups (*p*=0.045 and 0.019, respectively) (Figures 3(a) and 3(b)), but FeNO levels in both groups did not show significant changes before and after the study period (Figure 3(d)).

#### 3.2.3. Increased Asthma Control Scores

**Figure 4:**
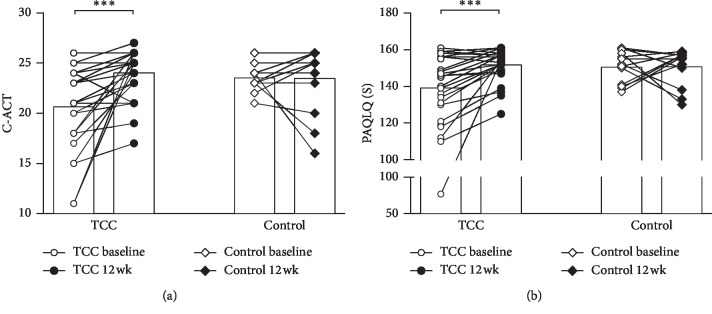
The symptoms of asthma surveyed using (a) the childhood asthma control test (C-ACT) and (b) the standardized pediatric asthma quality of life questionnaire (PAQLQ(S)) at baseline and 12 weeks after TCC. The bar denotes the mean level. ^*∗∗∗*^*p* < 0.001.

In the TCC group, but not the control group, a significant improvement in the C-ACT score after the 12-week training was observed (*p* < 0.001) (Figure 4(a)).

#### 3.2.4. Better Quality of Life

The overall PAQLQ(S) score improved significantly only in the TCC group (*p* < 0.001) and not in the control group (Figure 4(b)).

#### 3.2.5. Fewer Asthma Exacerbations Occurring after TCC Exercise

**Figure 5:**
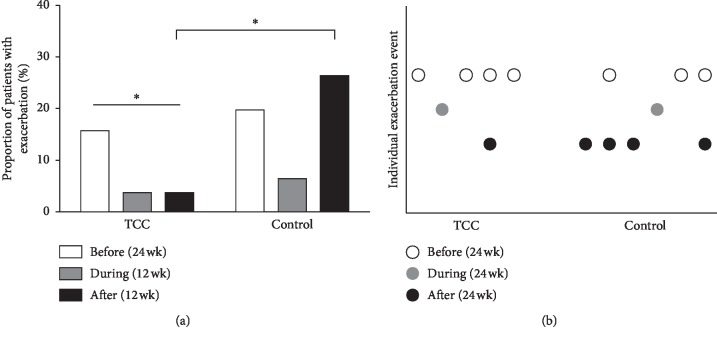
Exacerbations among study participants. (a) The proportion of patients with exacerbation 24 weeks before TCC, during TCC, and 12 weeks after TCC. ^*∗*^*p* < 0.05. (b) Individual exacerbation events occurring 24 weeks before TCC, during TCC, and 12 weeks after TCC. The same *X* value denotes the same patient.

The proportion of patients with exacerbations is shown in Figure 5. There was no difference between the TCC and control groups within 24 weeks before intervention. During the 12 weeks of TCC training, the proportion of patients with exacerbation decreased in both groups. Within 12 weeks after this study, the proportion of patients with exacerbation significantly decreased in the TCC group compared to the control group (*p*=0.031). There was no asthma-related emergency visit or hospital admission in the TCC group, but two asthma-related emergency visits were found in the control group. In addition, only the TCC group showed a significant decrease in the prevalence of exacerbation after the study period (*p*=0.019, before vs. after the study period). The individual exacerbation event is presented in Figure 5(b), indicating that most of the exacerbations (67%–80%) occurred in different patients. After multiple regression by a conditional forward method, the factors that were significantly associated with exacerbation within 12 weeks after the study is the practice of TCC and exacerbation within 24 weeks before the study (*p*=0.013 and 0.015, respectively) after adjusting for age, sex, asthma severity, PEF, FEV_1_, C-ACT, PAQLQ(S), and medication score at baseline.

### 3.3. Exercise Adherence

Of the 25 participants in the TCC exercise group, not all individuals managed to complete the 12-week in-person training program due to scheduling conflicts. Six participants had an attendance rate (actual training days/scheduled training days) of 74% or less, whereas seven participants had a 75%–90% attendance rate. Twelve participants attended more than 90% of the training sessions. Each participant in the TCC group was asked to practice TCC exercise at home once daily following a video demonstration, including the day of a missed training session.

For the TCC exercise at home, 23 out of 25 (96%) participants attended more than 75% of the study period. There were 5 participants (20%) exercising TCC every day with a 100% attendance rate.

## 4. Discussion

Our previous study investigating the effects of 12 weeks of TCC training on asthmatic and nonasthmatic children showed that the level of FeNO and PEFR and the FEV_1_/FVC ratio improved significantly in both groups after TCC. The asthmatic children also had improved QoL after TCC [17]. Given the evidence, we would like to further survey the effects of TCC on asthmatic children and potential mechanisms. We found that the group of children who chose to participate in the TCC group had improved pulmonary functions by the end of the study relative to the children who chose not to participate in TCC. Our results are in agreement with the literature, showing that children with asthma had improved pulmonary functions (including FVC, FEV_1_, and PEFR) following TCC exercise [16]. However, there are still conflicting reports on whether or not physical activity enhances pulmonary functions in healthy or asthmatic individuals [5, 24, 25]. These discrepancies are most likely due to the frequency, duration, and types of exercise sessions performed in individual studies. One study has indicated that the lack of significant improvements in pulmonary function might be a result of structural change/modeling of the lung in asthmatic individuals [26]. The TCC group had increased FVC by the end of the study compared to baseline, which was not found in the control group, suggesting that the increase in FVC might be a result of the breathing techniques incorporated into the TCC exercise, improving the participant's lung capacity.

There were a limited number of studies using validated instruments such as the C-ACT or ACT to assess the clinical control of asthma. Our study used both the C-ACT and indirect measures (i.e., exacerbations and frequency of emergency visits and/or hospitalization) to assess the effect of TCC exercise on asthma control. The results revealed that children in the TCC group had better C-ACT scores and fewer exacerbations, while the medication scores were not different in the two groups. As expected, QoL among asthmatic children assessed from the PAQLQ(S) scores was also significantly improved after TCC exercise, which might be related to the improved asthma control. These favorable effects of TCC observed in our study are also consistent with the findings in recent meta-analysis and systemic reviews, i.e., regular physical exercise has a positive effect on QoL in asthmatics, with evidence of improvements in bronchial hyperresponsiveness and FEV_1_ [5, 25, 27]. As few exacerbations which occurred during the study period (even in the control group) may be due to seasonal factors, the exacerbations of asthma usually peak in the fall and spring [28]. Since our study was conducted in summer, fewer exacerbations in both groups could be expected. In the control group, the frequency of exacerbation was increased within 12 weeks after the study as the fall arrived (Figure 5(a)). But such a phenomenon was not noted in the TCC group, suggesting that TCC exercise might prevent exacerbation in the peak season.

FeNO is a widely accepted noninvasive marker of airway inflammation in asthma. High FeNO in the breath of patients with asthmatic symptoms is correlated with high levels of airway eosinophils [29, 30]. Our previous study showed decreased FeNO in asthmatic and nonasthmatic children after TCC exercise. However, many demographic and biological factors, including sex, height, age, race-ethnicity, cigarette smoking, atopy, IgE levels, eosinophil count, and the time of day of the FeNO exam, may result in FeNO variations [31, 32]. In the present study, the levels of FeNO before and after the study period in both groups were found to have no significant difference, which may be due to the short period of TCC training or other factors described above.

Accumulating clinical data supports the notion that the immune system responds to increased physical activity in association with long-term antiinflammatory effects [33, 34]. Since asthma is characterized as a chronic airway inflammatory disease with immediate-phase allergic reactions [35] triggered by allergen stimulation and recruitment of inflammatory cells (including eosinophils, dendritic cells, macrophages, and T lymphocytes) [36], as well as the recruitment of T helper type 2 (Th2) lymphocytes in the late phase of the inflammatory response [37], it has been reported in adults that TCC exercise may be helpful in asthma control by enhancing innate and adaptive immunity (e.g., increased IgA, IgG, and IgM levels [38], increased CD3^+^ T cells, CD19^+^ B cells, and CD16^+^CD56^+^ NK cells, decreased CD3^+^ cytotoxic T-cell count [11], and an increase in the Th1 response [39]). At present, studies on the effects of TCC on immune system regulation among asthmatic children are scarce. In our study, it was found that 12 weeks of TCC exercise decreased the amount of total peripheral leukocytes and eosinophils and increased the percentage of CD4^+^CD25^+^Foxp3^+^ Treg cells. The increased Treg cells and Th1 responses (e.g., decrease in eosinophils) [39] after TCC training may counteract Th2 inflammation in asthma and therefore improve lung functions, providing better asthma control and decreased exacerbations.

Several limitations were identified in this study. First, this was not a randomized study. The patients chose to do TCC on their own will. Second, our study was limited by the short period of observation and a relatively small number of participants. Also, the participants in the TCC group were trained once weekly by the instructor during the TCC session and they practiced at home following the provided video recordings. The quality of the TCC training at home cannot be guaranteed. Finally, the dropout rate was high in the control group (46.4%) while a lower dropout rate was noted in the TCC group (13.8%). In the dropout patients, there were 8 boys and 5 girls in the control group and exclusively 4 girls in the TCC group. The age was slightly higher in the dropout control group compared to the dropout TCC group (9.4 ± 0.6 vs 8.0 ± 0.8 years old). The BMI was increased in the dropout control group compared to the dropout TCC group (18.9 ± 1.1 vs 15.5 ± 1.6 kg/m^2^). The participants' BMI in our study is between the 50^th^ and 75^th^ percentile, while the dropout control participants' BMI is around the 85^th^ percentile and the dropout TCC participants' BMI is around the 50^th^ percentile. In the dropout control group, there were 7 patients with mild persistent asthma, 2 with moderate persistent asthma, and 4 with severe persistent asthma. In the dropout TCC group, there were 2 patients with mild persistent asthma and 2 with severe persistent asthma. Less than half of the dropout patients received baseline laboratory and lung function tests and questionnaires. We are not sure that the higher-BMI participants in the dropout control group could benefit from TCC since they were prone not to practice TCC (and even other exercises). Further studies of TCC exercises in obese and normal weight patients may be needed to survey the immune modulation effect of TCC in higher-BMI patients. Despite all the limitations, our study still provided valuable clinical information.

## 5. Conclusions

Our study suggests that integrating TCC exercise into an asthma treatment plan may be beneficial for asthmatic children, and 12 weeks of TCC training could improve pulmonary functions, regulate immune function, provide better asthma control, improve QoL, and finally prevent asthma exacerbations. Further randomized studies with a larger sample size are needed to substantiate current findings.

## Figures and Tables

**Table 1:** Characteristics of the Tai Chi Chuan (TCC) and control group.

Characteristics	TCC (*n* = 25)	Control (*n* = 15)	*p* value
Sex (*n*, %)			
Male	14 (56.0%)	7 (46.7%)	0.567
Female	11 (44.0%)	8 (53.3%)	
Age (years)	8.3 ± 0.3 (6.9–9.2)	8.0 ± 0.3 (6.9–9.0)	0.675
BW (kg)	28.3 ± 1.7 (21.6–34.0)	25.6 ± 1.2 (20.8–29.4)	0.418
BH (m)	1.281 ± 0.021 (1.210–1.305)	1.257 ± 0.018 (1.200–1.320)	0.695
BMI (kg/m^2^)	16.9 ± 0.6 (14.6–20.3)	16.2 ± 0.7 (14.2–17.9)	0.442
Allergic rhinitis history	22 (88.0%)	14 (93.3%)	0.586
Atopic dermatitis history	4 (16.0%)	0 (0.0%)	0.102
Asthma severity			
Mild persistent	13 (52.0%)	9 (60.0%)	0.377
Moderate persistent	8 (32.0%)	2 (13.3%)	
Severe persistent	4 (16.0%)	4 (26.7%)	
Daily medication scores	2.8 ± 0.5 (1.0–4.1)	3.2 ± 0.9 (0.0–6.0)	0.932
Admission in the past 6 months	2 (8.0%)	1 (6.7%)	0.877
Exacerbation in the past 6 months	4 (16.0%)	3 (20.0%)	0.747

Data shown are mean ± SE (interquartile range) or the number (%) of patients as appropriate. BW, body weight; BH, body height; BMI, body mass index.

**Table 2:** The laboratory and lung function tests and questionnaires of the Tai Chi Chuan (TCC) and control group at baseline.

	TCC (*n* = 25)	Control (*n* = 15)	*p* value
Leukocytes (/*μ*L)	9083.6 ± 491.7 (7480–10430)	8006.7 ± 385.3 (6960–9200)	0.204
Eosinophils (/*μ*L)	552.8 ± 81.4 (295.4–663.5)	347.1 ± 72.4 (118.8–510.6)	0.067
Treg percentage of CD4 (%)	1.95 ± 0.16 (1.30–2.40)	2.07 ± 0.41 (1.41–2.39)	0.824
IgE (IU/mL)	577.4 ± 131.4 (133.3–755.0)	503.4 ± 111.2 (50.2–861.0)	0.966
FEV_1_ (L)	1.38 ± 0.06 (1.22–1.48)	1.39 ± 0.08 (1.20–1.67)	1.000
FEV_1_ predicted (%)	89.9 ± 3.1 (80.0–98.6)	92.3 ± 3.3 (83.0–101.5)	0.564
PEFR (L/min)	306.4 ± 13.8 (265.5–349.0)	321.1 ± 17.5 (280.5–352.5)	0.447
PEFR predicted (%)	78.2 ± 4.0 (64.5–82.6)	81.2 ± 3.0 (75.1–90.0)	0.329
FVC (L)	1.60 ± 0.08 (1.32–1.78)	1.63 ± 0.12 (1.26–2.12)	0.952
FVC predicted (%)	87.7 ± 3.0 (77.5–96.8)	92.0 ± 5.3 (77.0–101.0)	0.627
FeNO (ppb)	13.8 ± 2.1 (6–19.5)	14.3 ± 3.7 (4–26)	0.595
C-ACT	20.6 ± 0.9 (18–24)	23.5 ± 0.4 (23–24)	**0.045**
PAQLQ(S)	139.3 ± 3.9 (130.5–156)	150.0 ± 2.2 (141–158)	0.065

Data shown are mean ± SE (interquartile range). Treg, regulatory T cells; IgE, immunoglobulin E; FEV_1_, forced expiratory volume in one second; predicted (%), the percentage of predicted value according to the age, sex, body weight, and height with reference from the Ministry of Health and Welfare in Taiwan; PEFR, peak expiratory flow rate; FVC, forced vital capacity; FeNO, fractional exhaled nitric oxide; C-ACT, childhood asthma control test; PAQLQ(S), the standardized pediatric asthma quality of life questionnaire. Reference range: leukocytes, 4000–10500/*μ*L; eosinophils, 50–250/*μ*L; IgE, <100 IU/mL; FeNO, a higher level correlates with airway inflammation: <20 ppb as low, 20–30 ppb as moderate, and >35 ppb as high concentration; C-ACT, a higher score reflects better controlled asthma: ≥20 as well controlled and ≤19 as not well controlled; PAQLQ(S), a higher score reflects better quality of life.

## Data Availability

The data used to support this report are available upon request from the corresponding author. As the participants involve children who are potentially identifiable from the full dataset, requests are subject to review by the Institutional Review Board of National Taiwan University Hospital, Taipei, Taiwan.
